# Taxonomy of the burden of treatment: a multi-country web-based qualitative study of patients with chronic conditions

**DOI:** 10.1186/s12916-015-0356-x

**Published:** 2015-05-14

**Authors:** Viet-Thi Tran, Caroline Barnes, Victor M. Montori, Bruno Falissard, Philippe Ravaud

**Affiliations:** Department of General Medicine, Paris Diderot University, 16 rue Henri Huchard, 75018 Paris, France; Centre de Recherche en Epidémiologie et Statistiques, INSERM U1153, 1 place du Parvis Notre Dame, 75004 Paris, France; Centre d’Épidémiologie Clinique, Hôpital Hôtel-Dieu, Assistance Publique-Hôpitaux de Paris, 1 place du Parvis Notre Dame, 75004 Paris, France; Paris Descartes University, 12 Rue de l’Ecole de Médecine, 75006 Paris, France; Division of Health Care and Policy Research, Department of Health Sciences Research and Knowledge and Evaluation Research Unit, Mayo Clinic, 200 1st St SW, Rochester, MN 55905 USA; Paris Sud University, 15 Rue Georges Clemenceau, 91400 Orsay, France; INSERM U669, 97 Boulevard de Port Royal, 75679 Paris, France; Department of Epidemiology, Columbia University Mailman School of Public Health, 116th St & Broadway, New York, NY 10027 USA

**Keywords:** Qualitative research, Chronic conditions, Cost of illness, Burden of treatment, Internet study

## Abstract

**Background:**

Management strategies for patients with chronic conditions are becoming increasingly complex, which may result in a burden of treatment for patients. To develop a Minimally Disruptive Medicine designed to reduce the burden of treatment, clinicians need to understand which healthcare tasks and aggravating factors may be responsible for this burden. The objective of the present study was to describe and classify the components of the burden of treatment for patients with chronic conditions from the patient’s perspective.

**Methods:**

We performed a multi-country qualitative study using an online survey and a purposive sampling strategy to select English-, French-, and Spanish-speaking participants with different chronic conditions. Participants were recruited by physicians, patients’ associations, advertisement on social media, and ‘snowballing’. The answers were analyzed by i) manual content analysis with a grounded theory approach, coded by two researchers, and ii) automatic textual analysis by Reinert’s method.

**Results:**

Between 2013 and 2014, 1,053 participants from 34 different countries completed the online survey using 408,625 words. Results from both analyses were synthesized in a taxonomy of the burden of treatment, which described i) the tasks imposed on patients by their diseases and by their healthcare system (e.g., medication management, lifestyle changes, follow-up, etc.); ii) the structural (e.g., access to healthcare resources, coordination between care providers), personal, situational, and financial factors that aggravated the burden of treatment; and iii) patient-reported consequences of the burden (e.g., poor adherence to treatments, financial burden, impact on professional, family, and social life, etc.). Our findings may not be applicable to patients with chronic conditions who differ from those who responded to our survey.

**Conclusions:**

Our taxonomy of the burden of treatment, provided by patients with chronic conditions from different countries and settings, supports the development of tools to ascertain the burden of treatment and highlights potential targets for interventions to minimize it.

**Electronic supplementary material:**

The online version of this article (doi:10.1186/s12916-015-0356-x) contains supplementary material, which is available to authorized users.

## Background

Multimorbidity is becoming increasingly common: 42 % of patients have one chronic condition and 23 % have multiple chronic conditions [1]. Further, multimorbid patients and their clinicians may struggle to balance the benefits and risks of multiple recommended treatments [2]. Indeed, physicians may be tempted to focus on individual diseases and follow clinical practice guidelines dedicated to one condition, but this approach may lead to overtreatment and unintended consequences [3, 4]. For example, a physician following clinical practice guidelines could prescribe up to 12 medications for a patient with osteoporosis, osteoarthritis, diabetes mellitus, hypertension, and chronic obstructive pulmonary disease [5]. As a result, in addition to the burden of illness, patients are affected by the burden of treatment, defined as the impact of the ‘work of being a patient’ on functioning and well-being [6]. This work includes drug management, self-monitoring, visits to the doctor, laboratory tests, lifestyle changes, and other actions that take place in addition to the other work patients and their caregivers must do as part of life [7, 8]. Coping with all these healthcare tasks requires a significant amount of additional time, effort, and cognitive effort from patients and caregivers and is associated with poor adherence to therapeutic care, independent of illness [9, 10].

The burden of treatment depends on patients’ investment of time and effort in following their physicians’ advice and on their context (e.g., social or family structure, care delivery system, etc.). This fact limits the transfer of findings from previous qualitative studies of specific conditions [11, 12] or in specific countries [7, 13]. Indeed, patients with similar conditions and treatment regimens could have different burdens of treatment depending on their education, culture, beliefs, family and social support, financial capacities, formal and informal support resources available, and healthcare context.

In the present study, we aimed to explore, describe, and classify the components and consequences of the burden of treatment for patients with at least one chronic condition, across multiple conditions, treatments, countries, and settings.

## Methods

We performed a qualitative study using open-ended questions in an online survey to explore patients’ experiences and difficulties in managing their chronic conditions in everyday life. The Internet tool consisted of a website describing the concept of burden of treatment and an online questionnaire in three different languages (English, French, and Spanish) (Additional file 1).

### Sample and recruitment

We recruited adult participants (>18 years old) with at least one chronic condition (defined as a condition requiring healthcare for at least 6 months) in three different ways: i) invitation by participating physicians, ii) invitation by participating patient associations, and iii) advertisement on popular online health forums and social media (Additional file 2). Participants who had been invited were encouraged to invite relatives and friends who had chronic conditions to participate by a ‘snowball’ sampling method [14], which involves identifying an initial number of participants who serve as ‘seeds’ to help identify peers who, in turn, are asked to invite others and so forth [15]. This sampling strategy was not designed to be representative of the population of patients with chronic conditions but rather sought to select a broad range of participants likely to experience different burdens of treatment.

All participants gave electronic informed consent before participating in the study. The study was reviewed and approved by the Institutional Review Boards of Cochin Hospital in France (no. 00001072) and the Mayo Clinic (Rochester, MN, USA).

### Data collection

In addition to demographic and clinical information, we collected qualitative data about patient experiences in managing chronic conditions in everyday life with open-ended questions.

First, we identified, in the literature, different aspects that contributed to the burden of treatment: taking medicines, self-surveillance (e.g., patients taking their blood pressure or measuring their blood sugar themselves, etc.), laboratory tests, doctor visits, learning about conditions and treatments, need for organization, transportation, administrative tasks, financial costs of treatment, difficulties in following advice on diet and physical exercise, the social impact of treatment, and problems associated with health organization (i.e., insurance coverage, access to care close to home, health policies, etc.) [7, 8, 11, 12, 16–19].

Second, we developed a preliminary questionnaire consisting of four parts: i) demographic and clinical information about the participant’s conditions and treatments; ii) a broad open-ended question at the beginning of the questionnaire to elicit the participant’s view of the burden of treatment; iii) 16 open-ended questions about the aspects identified from the literature; and iv) a broad-ended question at the end of the questionnaire to identify other aspects of the burden of treatment that could have an impact on patients’ quality of life but had not been assessed in the previous questions.

Third, this preliminary questionnaire was reviewed by seven physicians with experience in care of chronic conditions and pilot-tested, in pen-and-paper form, with 44 patients with chronic conditions recruited in university hospitals in Paris, in November 2012, to assess the clarity and wording of questions and types of answers. The final questionnaire was implemented online [20]. The ease of use and clarity of the Internet version were assessed by six patients and two physicians. All texts were translated into English and Spanish by professional translators and assessed by four native-speaking patients and investigators.

### Data analysis

Quantitative data are described with means (SD) for continuous variables and proportions for categorical variables.

Qualitative data were analyzed by content analysis and automatic textual analysis. First, we analyzed all participants’ responses using content analysis [21] with a grounded theory approach [22] to identify, for each patient, components and consequences of the burden of treatment. This analysis involved three steps. In a first step, two investigators (VTT and CB) independently identified for the first 200 responses in French and English, “in vivo codes”: literal terms used by participants to explain and describe their burden of treatment. During meetings, the investigators reached consensus on the initial codes and grouped them into an initial set of themes that seemed meaningful to participants. Consensus was informed by the investigators’ previous works on the burden of treatment [8, 9], the literature [7, 8, 11, 12, 16–19], and their clinical experience. In a second step, this initial set of themes was used for analysis of the remaining responses: each participant’s response was read by two investigators (at least one researcher native in the given language), who independently assigned data segments to each theme. Analyses involved untranslated data: participants’ verbatim answers were read in their original language, but coding was in English. During frequent meetings, the investigators compared their analyses and reached consensus on coding. Whenever a new idea emerged, researchers discussed the idea, thereby refining and enriching the list of themes. In a third step, an investigator (VTT) re-read participants’ contributions to assess consistency with the coding scheme.

Second, we examined the combined text of responses from all participants, for each language, using Reinert’s automatic textual analysis method [23–26]. This method is a meaning-blind automatic textual analysis relying on the assumption that a text contains a reciprocal relationship between words and their proximate environments. For example, a section of text about health would include words related to health, and words related to health would be indicative of sections of text about health. Therefore, general ideas of a text could be revealed through the internal organization of the text. Reinert’s method involves four steps: i) creation of a basic vocabulary dictionary by identifying lexical forms (i.e., nouns, verbs, adjectives, and adverbs) contained in the corpus; ii) fragmentation of the corpus into small parts, or elemental context units, consisting of approximately 10 to 15 words delimited by punctuation; iii) creation of a table of lexical forms and elemental context units; and iv) partition of the table into classes, using hierarchical descendant classification to group sections of texts according to their similarity (presence or absence) regarding the words in the texts using *χ*^2^ tests. Analysis of words for each class allows for identification of topics covered in a text. The number of clusters was arbitrarily defined to contain at least 100 sections of text. Automatic textual analysis involved the use of IRaMuTeQ 0.6 alpha 3 [27].

### Creation of a taxonomy of the burden of treatment

We developed a taxonomy using methods described by Bradley et al. [28]. In a first step, two investigators (VTT, CB) used the different themes identified during the manual analysis to delineate an initial classification. In a second step, they used results from the automatic textual analysis to refine the classification. Finally, several meetings were held between researchers to discuss the creation of the taxonomy in light of previous studies about the burden of treatment.

### Relationships between burden of treatment and respondent characteristics

To understand the context of each component of the burden of treatment, we examined the relationships between the patient’s statement of a specific burden and clinical variables. Logistic regression was used to summarize these relationships. Odds ratios (ORs) were adjusted for key confounding factors (age, sex, presence of multimorbidity, and educational level). We performed sensitivity analyses by using two definitions of multimorbidity (two or more and three or more chronic conditions). Indeed, multimorbidity defined as patients with more than two chronic conditions may lack specificity because of the high proportion of patients involved [29]. Some authors argued that using more than three disease entities would likely identify patients with greater health needs and would therefore be more useful to clinicians [30]. Analyses involved use of SAS 9.3 (SAS Institute, Cary, NC, USA).

## Results

**Fig. 1:**
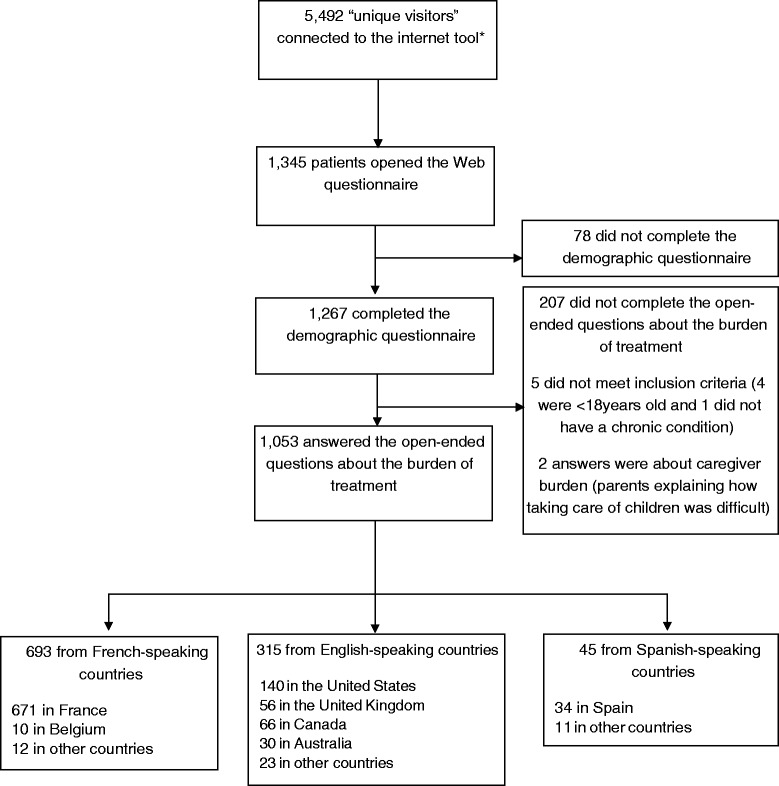
Flow of patients in the study. * “Number of unique visitors” from May 22, 2013 to March 30, 2014, assessed by use of Google Analytics. “Unique visitors” can include study participants, non-eligible patients, physicians, researchers, members of patient associations, or curious visitors. Details on the method of calculating the number of users can be found at https://support.google.com/analytics/answer/2992042?hl=en

From June 22, 2013, to March 30, 2014, 5,492 people connected to the Internet tool, and 1,345 people (24 %) identified themselves as eligible for the study; 1,267 (94 %) completed the demographic and clinical part of the survey, and 1,053 (78 %) answered the open-ended questions about the burden of treatment (Fig. 1). The mean (SD) age was 46 years and 766 (73 %) were women (Table 1). In total, 671 patients resided in France (64 %), 140 in the United States (13 %), 66 in Canada (6.3 %), 56 in the United Kingdom (5.3 %), 34 in Spain (3.2 %), 30 in Australia (2.8 %), and 56 (5.3 %) in a different country. Self-reported chronic conditions included rheumatologic diseases (33 %), cancer (8 %), and well-controlled psychiatric illnesses (13 %). The mean (SD) number of chronic conditions was 2.4 (1.6, range 1–10). A total of 662 patients (63 %) had two or more chronic conditions.

**Table 1:** Characteristics of participants (n = 1,053)

Characteristics	Total	Participants from French-speaking countries	Participants from English-speaking countries	Participants from Spanish-speaking countries
	(n = 1,053)	(n = 693)	(n = 315)	(n = 45)
Age (yr), Median (IQR)	47 (35–57)	46 (35–56)	49 (36–59)	45 (37–55)
Female sex, n (%)	768 (73)	474 (68)	262 (83)	30 (67)
Marital status, n (%)				
Married or civil union	544 (52)	352 (51)	170 (54)	22 (49)
Live in partner	129 (12)	98 (14)	28 (9.0)	3 (6.7)
Single	252 (24)	166 (24)	70 (22)	16 (36)
Divorced	107 (10)	63 (9.0)	42 (14)	2 (4.4)
Widowed	21 (2.0)	14 (2.0)	5 (1.6)	2 (4.4)
Highest educational level, n (%)				
Primary/middle school	30 (2.8)	23 (3.3)	6 (1.9)	1 (2.2)
High school	305 (29)	249 (36)	44 (14)	12 (27)
College	718 (68)	421 (61)	265 (84)	32 (71)
Place where participants go most frequently for consultations, n (%)				
Public hospital	465 (44)	378 (54)	65 (21)	22 (49)
Private hospital	83 (7.9)	61 (8.8)	17 (5.4)	5 (11)
General practitioner’s clinic	295 (28)	163 (24)	124 (39)	7 (16)
Specialist clinic	210 (20)	91 (13)	109 (35)	11 (24)
Presence of informal caregiver ^a^, n (%)	478 (45)	329 (47)	126 (40)	23 (51)
No. of medical appointments/month, Median (IQR)	4 (2–8)	5 (3–9)	3 (2–5)	3 (2–7)
No. of different physicians the participant regularly sees, n (%)	3 (2–4)	3 (2–4)	3 (2–4)	3 (2–4)
No. of tablets/day, Median (IQR)	8 (4–12)	7 (4–12)	8 (4–15)	4.5 (2–9.5)
No. of injections/day, Median (IQR)	0 (0–1)	0 (0–1)	0 (0–1)	0 (0–1)
Time needed to organize medications per week (minutes), Median (IQR)	30 (14–70)	21 (10–45)	35 (15–90)	35 (10–105)
Time needed for self-monitoring per week (minutes), Median (IQR)	8.5 (0–20)	5 (0–15)	10 (0–35)	17.5 (5–60)
No. of conditions, Median (IQR)	2 (1–3)	2 (1–3)	2 (1–4)	2 (1–3)
Participants with >2 chronic conditions, n (%)	662 (63)	409 (59)	224 (71)	29 (64)
Main chronic conditions ^b^				
Diabetes	168 (16)	98 (14)	53 (17)	17 (38)
Other endocrine disorders	197 (19)	106 (15)	81 (26)	10 (22)
Lung diseases	142 (13)	52 (7.5)	84 (27)	6 (13)
High blood pressure or dyslipidemia	303 (29)	207 (30)	87 (28)	9 (20)
Heart diseases	125 (12)	57 (8.2)	64 (20)	4 (8.9)
Kidney or urological diseases	304 (29)	285 (41)	18 (5.7)	1 (2.2)
Gastrointestinal diseases	138 (13)	66 (9.5)	66 (21)	6 (13)
Stroke or cerebrovascular disease	35 (3.3)	20 (2.9)	15 (4.8)	0 (0)
Neurologic diseases	146 (14)	89 (13)	49 (16)	8 (18)
Rheumatologic disease	344 (33)	209 (30)	127 (40)	8 (18)
Cancer or malignant blood diseases	84 (8.0)	59 (8.5)	21 (6.7)	4 (8.9)
Psychiatric disease	128 (13)	56 (8.1)	60 (19)	12 (27)
Vision problems	79 (7.5)	46 (6.6)	29 (9.2)	4 (8.9)
Otorhinolaryngological problems	64 (6.1)	42 (6.1)	21 (6.7)	1 (2.2)
Skin diseases	89 (8.4)	58 (8.4)	21 (6.7)	10 (22)
Infectious disease	12 (1.0)	8 (1.1)	3 (0.9)	1 (2.2)
Systemic conditions	108 (10)	70 (10)	38 (12)	0 (0)
Other ^c^	50 (4.7)	26 (3.7)	20 (6.3)	4 (8.9)

^a^ Informal caregivers were family members or friends who helped the participant with healthcare tasks without payment for the care; ^b^ A patient can have multiple chronic conditions; ^c^ Other included non-malignant hematological conditions, thrombosis, obstetrical conditions, genetic disorders

Answers to open-ended questions formed an overall corpus of 408,625 words, in English (148,707 words), French (243,558 words), and Spanish (16,360 words). Mean (SD) length of patients’ answers were 388 (359) words globally (maximum 2,699) and 108 (120) words for the first question (maximum, 1,267).

**Fig. 2:**
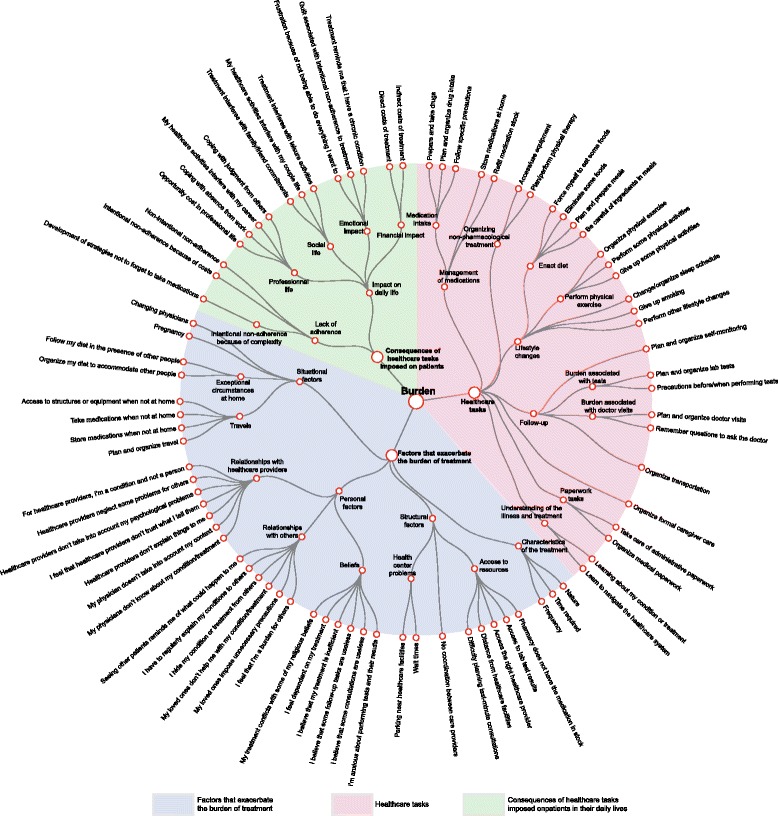
Taxonomy of the burden of treatment

Manual content qualitative analysis and automatic textual analysis provided coherent results and described a list of difficulties patients could have when performing healthcare-related tasks. These two analyses were synthesized in light of the literature to construct a taxonomy of the burden of treatment. This taxonomy described i) the tasks imposed on patients by their diseases and by their healthcare system, ii) the factors that exacerbate the burden associated with these tasks, and iii) how these tasks affected patients’ lives (Fig. 2).

### Manual content qualitative analysis

For clarity, results of the manual qualitative analysis are presented according to the final classification.

#### Healthcare tasks imposed on patients

Most patients acknowledged that the care associated with having a chronic condition imposed a number of extra tasks (Additional file 3): “*Being sick also adds a lot of extra tasks, paperwork and appointments. To keep myself healthy, I miss out on a lot of things that people my age take for granted – working fulltime, cooking, showering every day, going out to socialize*” (25-year-old woman from the United Kingdom with Ehlers-Danlos syndrome). These tasks could be classified as i) managing medications, ii) lifestyle changes, iii) condition and treatment follow-up, iv) paperwork tasks, and v) learning about the condition and treatment (Box 1).

##### Managing medications

Managing medications encompassed all tasks related to i) medication intake and necessary precautions before or during intake, ii) adaptation and planning of daily life to take medications, and iii) ensuring medication stock (e.g., refills, carrying medication at all times). Implementation of ‘simple’ prescriptions could sometimes result in major investments of time, energy, or cognitive effort from patients. For example, “*Doctors often forget the time it takes to do a treatment isn’t just the treatment itself. A nebulizer may finish in 6 minutes but that doesn’t include mixing the drug and cleaning and sterilizing the equipment. That bumps the time to 20 min and doing that up to 5 times a day is suddenly a large chunk of your day*” (28-year-old woman from the United Kingdom with a pulmonary and a gastrointestinal condition). In total, 676 participants (64 %) expressed at least one burden related to management of medication.

##### Lifestyle changes

Lifestyle changes referred to the efforts patients made to follow their physicians’ recommendations to i) avoid eating certain foods (including paying attention to ingredients or having to plan and prepare their own meals), drinking alcohol, or smoking; or ii) practice or give up physical exercise. For most participants, lifestyle changes were considered the most difficult tasks. For example, “*This makes me feel socially isolated. I cannot share much food with others, have to bring my own food, must deal with the hassle of having to explain to others why I cannot eat whatever I want, have the stigma of feeling weird and different from other people*” (57-year-old woman from the United States with pulmonary, dermatologic, and rheumatologic conditions)*.* In our study, 594 participants (56 %) expressed at least a burden related to lifestyle changes.

##### Condition and treatment follow-up

Condition and treatment follow-up referred to all doctor visits, lab tests, and complementary exams and self-monitoring that patients had to organize, schedule, and attend. The burden associated with these tasks was closely intertwined with structural factors (e.g., coordination between healthcare providers, access to health resources). For example, “*For many appointments, you must leave time for: getting to the appointment, finding parking, waiting for the appointment, seeing the doctor, getting back home. That can easily wipe out a morning or an afternoon”* (75-year-old Canadian woman with thyroid disease, high blood pressure, and a rheumatologic condition)*.* Overall, 527 participants (50 %) expressed at least a burden associated with tasks of condition/treatment follow-up.

##### Paperwork tasks

Paperwork tasks required time and cognitive effort for patients. They had to learn, understand, and deal with complex rules and requirements necessary for their care and reimbursements. For example, “*It is difficult to fill these kinds of forms by yourself as there is a special language/code – there are certain things you have to say and certain things you have to avoid saying. It is sometimes very difficult to get help from a social worker or someone else who knows how these things work*” (28-year-old woman from the United Kingdom with cystic fibrosis). A total of 304 participants (29 %) expressed at least a burden associated with paperwork tasks.

##### Learning about and developing an understanding of the imposed tasks

Learning and developing an understanding of the imposed tasks was cited as a mandatory task for patients with chronic conditions. It involved making sense and keeping up to date of medical jargon and often-conflicting information from different sources (e.g., relatives, Internet, nurses, physicians) before implementing this knowledge into their lives. For example, “*When I first started to take all the different medications it was completely overwhelming, learning how each tablet interacted with another one, how some had to be taken with food, some 1 hour before food, some 1 hour after food, some not within 2 hours of another one, some tablets can only be taken 12 hours apart, it can seem ridiculous until you get into a routine*” (40-year-old Australian woman with endocrine and renal disorders). In all, 133 participants (13 %) expressed at least a burden associated with making sense of everything that was asked of them.

#### Factors that exacerbate the burden of treatment

The burden of healthcare tasks imposed on patients could be exacerbated by i) the nature, time required, and frequency of the imposed tasks, as well as by ii) structural, iii) personal, iv) situational, and v) financial factors (Box 2).

##### Nature, time required, and frequency of healthcare tasks

The nature (e.g., size, taste of tablets, discomfort associated with injections or blood tests, side effects of medications, life-long treatment duration), time required, and number and frequency of tasks imposed on patients were frequently cited as an aggravating factor of the burden of treatment (Additional file 4). For example, “*It is slow to take tablets – cannot take too many at a time because of gagging/nausea. Also some have a very strong bitter taste – on days when my hands are not working well it is difficult to get them in, sip water, and swallow quickly*” (39-year-old Chinese woman with an auto-immune condition). In our study, 641 (61 %), 422 (40 %), and 638 (60 %) participants expressed that their burden of treatment was exacerbated by its nature, the time required, and the frequency of the imposed tasks, respectively.

##### Structural factors

Structural factors depend on the healthcare system in the country of residence. They relate to i) poor access to healthcare resources (e.g., medication is not available at the pharmacy, distance to healthcare providers, difficulties in obtaining test results); ii) lack of coordination between care providers, who often care for patients individually without integrating their care in a more global process; iii) problems directly related to health centers (e.g., waiting times, lack of parking space); iv) lack of meaningful research on their conditions or treatments (e.g., including both lack of available research on specific rare chronic conditions and/or lack of knowledge of research in providers); and v) inadequate public coverage of their conditions or treatments (Additional file 5). For example, “*Trips to hospital at least every three months* [are] *burdensome because of distance and also parking charges. Annual reviews are difficult because there are multiple investigations required and these take place over multiple different appointments and sometimes at different hospitals in the same group. Organizing repeat prescriptions is difficult: GPs are monitored for their prescribing and most of my medication comes from my GP. GP is not allowed any flexibility over prescribing* […]*: this means multiple trips to GP and pharmacy every month*” (55-year-old woman from the United Kingdom with diabetes and a cardiac condition). Overall, 366 (35 %) participants told us that their burden was exacerbated by poor access to healthcare resources, 100 (9.5 %) commented on the lack of coordination between their healthcare providers, and 316 (30 %) expressed difficulties with health care centers.

##### Personal factors

Personal factors encompassed all factors related to i) patients’ beliefs about their conditions and treatment (e.g., feeling that their treatment lacked efficacy or that they were dependent on their medications); ii) the difficulties patients could have in their interactions with others (e.g., patients’ feeling that they were a burden on their relatives, patients’ desire to hide their condition/treatment from others), for example, “*I’m a burden for my family. I need more attention and care than my children*” (45-year-old French man with hemi-paretic stroke); and iii) difficulties patients could have in their interactions with healthcare providers, for example, “*I think it’s ridiculous that I have to do follow-ups every month when my doctor doesn’t appear to know much about my disease and definitely doesn’t listen to me when I’m telling him how I’m doing and doesn’t answer questions*” (33-year-old woman from the United States with cancer, a rheumatologic condition, and depression) (Additional file 6). A total of 252 (24 %) participants expressed that some beliefs about their conditions or treatment aggravated the burden associated with healthcare tasks; 402 (38 %) said that some interactions with others could exacerbate their burden of treatment and 230 (22 %) participants commented on how difficulties in their relationships with healthcare providers added to their burden of treatment.

##### Situational factors

Situational factors encompassed out-of-routine situations. Most participants, including those who had reconfigured their lives with their treatment and set up daily routines, cited the difficulties they had in performing healthcare-related tasks when they were away from home and/or in the presence of other people (Additional file 7). For example, “*Good grief* […] *I’m away on a vacation right now, and I had to go out the afternoon before we left to go buy more pill organizers, because I realized I didn’t have enough for the amount of time this trip was going to take*” (46-year-old woman from the United States with a neurological condition). In our study, 431 (41 %) participants expressed difficulties adapting to out-of-routine situations.

##### Financial factors

Financial factors could impede patients’ ability to do everything that was asked of them. These factors often were both aggravating factors and consequences of the burden of treatment. For example, “*I do not have insurance. I pay for every blood test, medication, doctor visit and the failed radioactive iodine uptake out of my own pocket. It is outrageously expensive and a tremendous burden”* (38-year-old man from the United States with a thyroid disease).

#### Consequences of healthcare tasks imposed on patients on their daily lives

Consequences of the aforementioned workload of healthcare involved i) poor adherence to the tasks imposed on patients; ii) impact on family, social, and professional life; iii) personal and emotional impact; and iv) financial impact of healthcare (Box 3 and Additional file 8).

##### Poor adherence to healthcare tasks

Poor adherence associated with the burden of treatment could be classified as intentional and non-intentional non-adherence. Intentional non-adherence involved patients giving up some tasks asked of them (from medication intake to administrative tasks) because of the additional financial costs or because it was “*too much*”. This non-adherence was different from intentional non-adherence, which follows from having a different understanding of disease and treatment that reduces concern for treating the disease or increases concern about the treatment’s safety or lack of efficacy. An example of intentional non-adherence due to treatment burden follows: “*There is stuff that I am supposed to do, and stuff that I actually do. If I did everything I am supposed to do, my life would revolve around doctors and tests and such and there wouldn’t be very much left for living my life. So I’ve made a bunch of choices*” (46-year-old woman from the United States with a mitochondrial disease). Non-intentional non-adherence refers to the difficulties patients had in remembering to take medications or attend appointments, especially with a multitude of imposed tasks. As a result, patients developed strategies not to forget taking their medications: i) use of pillboxes, ii) telephone reminders, iii) calendar, iv) daily actions or rituals, and v) help from relatives. In our study, 64 (6.1 %) participants reported a form of intentional non-adherence and 392 (37 %) expressed having problems not to forget to follow their treatments.

##### Impact on professional, social, family life, and leisure activities

Impact on professional, social, family life, and leisure activities was defined as consequences of healthcare on patients’ capacity to i) work, from the time taken by healthcare activities and the difficulties they had to arrange with their co-workers to keep up their work, to the judgment of colleagues or employers; ii) participate and live a family, social, or couple life; and iii) spend free time doing what they wanted. For example, “*Friendships are difficult to maintain because you end up cancelling plans and feel guilty every time you cancel on a friend or a family member. I would never wish a chronic illness on anyone*” (37-year-old Canadian woman with endocrine and rheumatologic conditions). In total, 555 (53 %) participants mentioned a negative consequence of healthcare activities on their professional, social, and family life.

##### Emotional impact

The emotional impact of the burden of treatment related to i) the frustration patients could experience by not being able to do everything they could have done because of their treatments; for example, “*Low cholesterol, gluten sensitive, low calorie – I love food but feel like I’m never really eating –* […] *I’m a prisoner to food*” (64-year-old woman from the United States with high blood pressure, vision problems, and cardiac and rheumatologic conditions); ii) the guilt patients could have when not following prescriptions or recommendations; and iii) regular healthcare, which reminded them that they had chronic conditions. Fr example, “*Medication intakes remind us that we have a lifelong incurable condition even if we look normal. It’s depressing*” (19-year-old French woman with systemic lupus). Overall, 325 (31 %) participants mentioned that their treatment was responsible for a negative emotional impact on their lives.

##### Financial impact

Financial impact of the treatment represented both direct costs of healthcare demands and indirect costs associated with treatment (e.g., losing a job, costs of specific foods). This burden greatly varied between contexts and was closely related to both structural factors and patients’ ability to deal with paperwork to get reimbursed. As stated earlier, financial factors were both consequences and aggravating factors of the burden of treatment, forming a vicious circle. For example, “*All my money goes on my health aside from basic bills. I do not buy treats, clothes, haircuts, toiletries, things for the house* […] *Have to spend a lot of time and energy on budgeting and I delay treatment sometimes as I have to save up*” (37-year-old woman from the United Kingdom with pernicious anemia and vision problems). In our study, 225 (21 %) participants mentioned a financial burden of their treatment.

### Automatic textual analysis

Automatic textual analysis was performed for each language separately. For English, we obtained 11 classes with at least 100 elemental context units, after descendant hierarchical classification. Dendograms and words strongly associated with each class (*χ*^2^ > 50) are given in Additional file 9.

The organization of text reflected the different tasks participants performed to take care of their health. Each class consisted of words describing the different aspects of each problem (e.g., blood-test, lab, visit, scan, self-monitor) and words highlighting the difficulties encountered (e.g., wait, time, rush, etc.).

During classification, we found a contrast between tasks directly related to healthcare that patients performed themselves (organize tests, appointments, refills, manage medications, and cope with side effects) and tasks that involved or had an impact on others, reflecting ‘out-of-medical-world interactions’ (lifestyle changes, relationships with family, friends, society, financial aspects of healthcare). Concerning the tasks patients directly related to healthcare, we found a distinction between medication and treatment management (side effects, strategies not to forget to take medications, and organization during travel) (23 % of the corpus) and ‘out-of-home’ care (tests, doctor visits, refills) (28 % of the corpus). Concerning tasks involving others out of the medical world, we found a distinction between paperwork and financial burden (14 % of the corpus), which were closely related, and lifestyle changes and relationships with family, friends, and other people (34 % of the corpus).

We found a similar organization of the corpus for ‘spontaneous answers’ (i.e., answers to the first open-ended question). For the two other languages, we found a greater emphasis on self-monitoring for tasks patients performed themselves (Additional file 10). This finding may be explained in part by the greater prevalence of conditions in which self-monitoring was integral to care, such as diabetes among Spanish-speaking respondents.

### Relationships between components of the burden of treatment and respondents’ characteristics

In our sample, participants with more than two chronic conditions more often elicited problems related to drug intake (OR = 1.7 [1.3–2.4]), paperwork tasks (OR = 1.4 [1.1–1.9]), and time required for tasks (OR = 1.4 [1.0–1.8]) than those with only one chronic condition. They more often felt like they were a burden to others (OR = 1.5 [1.0–2.2]), elicited more problems in their relationships with their healthcare providers (OR = 1.6 [1.2–2.3]), and expressed more adherence issues (OR = 2.2 [1.2–4]). When considering multimorbidity as patients with more than three chronic conditions, we found fewer differences between proportion of components of the burden of treatment identified by participants with or without multimorbidity, except for lack of adherence (Additional file 11). Older patients (>50 years old) less often elicited problems related to their professional life (OR = 0.34 [0.25–0.46]; Additional file 12). Female participants and those with a higher educational level (College) were more likely to elicit different components of the burden of treatment (Additional files 13 and 14).

## Discussion

With data from our qualitative study, we developed a comprehensive taxonomy of the burden of treatment for patients with chronic conditions in different contexts and countries. Independent analyses by informed investigators and a meaning-blind automatic procedure provided coherent results. Whereas manual qualitative analysis allowed us to explore the variety of themes mentioned by participants, automatic analysis allowed us to better understand how patients could dichotomize their lives between their life in hospitals or clinics and their lives outside of the medical world, the latter sometimes shrinking in favor of the former. In our study, patients mentioned a number of healthcare tasks imposed on them. The burden associated with these tasks could be aggravated by multiple personal, structural, or situational factors and affect quality of life and adherence to treatments, especially when consequences of treatment were not immediately visible or when treatment required extensive lifestyle changes. To our knowledge, this is the first study to provide a comprehensive view of the components and consequences of the burden of treatment across multiple countries, settings, and treatment context. Our findings fit the Cumulative Complexity Model [31] in that the burden of treatment perceived by patients was a complex phenomenon resulting from the combination of i) the workload of healthcare imposed on patients; ii) patients’ capacities to integrate this workload of healthcare in their daily lives in terms of energy, time, money, or support; and iii) the context, especially the structure of healthcare (i.e., travel distance for physician visits, waiting times, coordination between healthcare providers, reimbursements, etc.) and social support from their families, relatives, and friends.

In the present study, we assessed the relationships between patients’ statements of specific components of the burden of treatment and their characteristics. Multimorbid patients were more likely to express concerns about drug intake, time required for healthcare tasks, and talked significantly more often about adherence problems. These results agree with those from observational studies of the workload of care of multimorbid patients [8, 32] and highlight the magnitude of what patients perform unbeknownst to their physicians [32, 33]. If physicians spend about 2 hours each year with diabetic patients, these patients spend approximately 870 hours managing the disease on their own [34]. Because this involvement of time and effort is not usually discussed in depth during medical consultations [35], physicians should use adequate tools to diagnose and assess the burden of treatment [8].

The strength of this study relies in our focus on reducing the researcher’s impact on the analysis by involving multiple researchers in the manual content analysis, each researcher supplementing and contesting others’ statements [36], and by performing two separate analysis, one of which was automatic. Our results contribute to the body of literature describing the burden of treatment for patients with at least one chronic condition. Compared to the work of Gallacher et al. [11], we gathered comparatively more data from a larger number of diverse participants. Thus, our findings may have broader applicability to the general population of patients with chronic conditions. Differences between our two classifications result from conceptual choices: Gallacher et al. [11] used the processes patients use to implement tasks in their everyday life as key domains, whereas we used the tasks themselves. Similar to Eton et al. [7] and Sav et al. [13], we used, as key domains, the imposed tasks and aggravating factors of the burden of treatment. However, contrary to previous works, we integrated consequences of the burden of treatment in the taxonomy. Indeed, our analyses showed that patients indicated all aspects of the burden of treatment, including consequences, as a whole.

Our taxonomy of the burden of treatment also compliments the work with the Treatment Burden Questionnaire (TBQ) [8, 9]. Themes identified in the present study overlapped with the items of the TBQ but offered more precision and details, especially concerning the consequences and aggravating factors of the burden of treatment. This finding was not unexpected as the TBQ was developed to offer physicians the tools to identify overburdened patients, in practice. As a result, the tool was voluntarily short and concise. Our taxonomy of the burden of treatment offers solid qualitative bases for the development of specific and context-dependent measures that should complement generic measures of the burden of treatment, like the TBQ.

An original aspect of this study was the use of an online survey to gather data from geographically distant participants. Previous studies aiming to identify key concepts of a given topic in multiple countries were complex because they involved participation of researchers in each country [37]. In this study, we demonstrated the feasibility of using the Internet to gain insight into the experiences of patients directly and generate qualitative data simultaneously in multiple countries at low cost.

Nevertheless, this study has some limitations. First, we used an online questionnaire with organized in a rigid order with no adaptation to prior responses or in-depth probing for more specific information in response to patients’ statements. As a result, each patient’s contribution may be less rich than what could have resulted from an interactive face-to-face interview [38]. Thus, this situation represents a trade-off from the gain in diversity of respondents. Second, the questionnaire could have influenced participant responses (e.g., by providing a starting point rather than allowing the patient to truly respond from the ‘ground’ up). However, we found the exact same categories when considering only the first broad open-ended questions with subsequent questions allowing us to precise more fine grain themes. Third, the applicability of our findings may be limited given that our patients are not representative of the population of patients with at least one chronic condition in those countries (especially in Spanish-speaking countries, where we recruited only a small number of participants) or in the world. We only recruited patients using the Internet, which may have selected a population of respondents who were younger, more educated and ‘computer savvy’ [39]. For example, in our study, only one patient mentioned language barriers as a burden of treatment, although it is an important barrier to healthcare access [40, 41]. Similarly, we did not account for income or insurance coverage, which are likely associated with patient-reported burden of treatment. Given the proportion of patients with high educational level, participants with lower socioeconomic level were likely underrepresented in our sample. Because of the link between socioeconomic status and multimorbidity, the resource burdens and demands on patients’ financial capacity may have been underrepresented. Finally, the variation in how patients answered the questionnaire prevents from drawing conclusions other than the diversity of the components, aggravating factors, and consequences of the burden of treatment. Some patients may have not mentioned some of the difficulties with their treatments because of poor understanding of questions, recall problems, and/or social desirability bias [42]. For example, participants with a lower educational level were less likely to elicit components of the burden of treatment than those with higher educational level.

Our findings have several implications. First, our results may help clinicians better understand and identify patients who are overwhelmed by their treatments. In a previous study, we have shown that physicians fail to assess the burden of treatment of their patients [8], partly because it expresses a patient experience that is not shared in depth during consultations [35]. Second, our study points to the dire need to redesign guidelines to take into account multimorbidity [4, 43]. We found that multimorbid patients have more difficulties due to fragmentation of care; 60 % of patients had two or more chronic conditions. They expressed a significantly greater number of difficulties performing healthcare tasks, independent of structural factors, age, and country. Healthcare should be integrated and coherent: every therapeutic intervention imposed on patients should be carefully weighed in terms of clinical benefit, interaction with other treatments, possible harms, and potential burdens. Such consideration could result in the prioritization of tasks and a net reduction in healthcare tasks imposed on patients. Third, on a research level, new interventions should be designed to mitigate the aggravating factors identified, to improve patient adherence to treatment, and to reduce the unintended negative impact of such treatments, through the burden they impose on patients’ capacity, on their quality of life. Finally, on a structural level, this study highlights the need to change the paradigm of care for patients with chronic conditions and end fractured care focused on individual conditions. Treatment objectives should not be based solely on attaining specific goals in specific diseases but also on mitigating the impact of conditions and treatments on patients’ professional, family, and social lives [44], for minimally disruptive medicine.

## Conclusions

Data from our qualitative study of patients with different chronic conditions, in different contexts and countries, provides a comprehensive taxonomy of the burden of treatment for such patients. Results could inform the development of cross-cultural instruments to assess the burden of treatment for patients with chronic conditions and new interventions to reduce the burden of treatment, ultimately moving towards minimally disruptive medicine [6].

## Box 1: Healthcare tasks imposed on patients

**1.1. Management of medications**

- Prepare and take drugs
- Plan and organize drug intake
- Follow specific precautions before, during, or after medication intake
- Store medications at home
- Refill medication stock

**1.2. Organizing and performing non-pharmacological treatment**

- Access/use equipment
- Plan/perform physical therapy

**1.3. Lifestyle changes**

- Force myself to eat some foods
- Eliminate some foods
- Plan and prepare meals
- Be careful of ingredients in meals
- Organize physical exercise
- Perform some physical activities
- Give up some physical activities
- Change/organize sleep schedule
- Give up smoking
- Perform other lifestyle changes

**1.4. Condition and treatment follow-up**

- Plan and organize self-monitoring
- Plan and organize lab tests
- Precautions before/when performing tests
- Plan and organize doctor visits
- Remember questions to ask the doctor
- Organize transportation

**1.5. Organize formal caregiver care**

**1.6. Paperwork tasks**

- Take care of administrative paperwork
- Organize medical paperwork

**1.7. Learning and developing an understanding of the illness and treatment**

- Learning about my condition or treatment
- Learn to navigate the healthcare system

## Box 2: Factors that exacerbate the burden of treatment

**2.1. Nature, time required, and frequency of healthcare tasks**

**2.2. Structural factors**

- Access to resources
  - o Pharmacy does not have the medication in stock
  - o Access to lab test results
  - o Access the right healthcare provider
  - o Distance from healthcare facilities
  - o Difficulty planning last-minute consultations
- No coordination between care providers
- Health center problems (e.g., wait times, parking near healthcare facilities)
- Not enough research done on my condition
- Insufficient or inadequate media coverage of my condition

**2.3. Personal factors**

- Beliefs
  - o I’m anxious about performing tests and their results
  - o I believe that some consultations are useless
  - o I believe that some follow-up tasks are useless
  - o I believe that my treatment is inefficient
  - o I feel dependent on my treatment
  - o My treatment conflicts with some of my religious beliefs

- Relationships with others (except healthcare providers)
  - o I feel that I’m a burden for others
  - o My loved ones overdo things/impose unnecessary precautions
  - o My loved ones don’t help me with my condition/treatment
  - o I hide my condition or treatment from others
  - o I have to regularly explain my conditions to others
  - o Seeing other patients reminds me of what could happen to me in the future

- Relationships with healthcare providers
  - o My physicians don’t know about my condition/treatment
  - o My physician doesn’t take into account my context
  - o Healthcare providers don’t explain things to me
  - o I feel that healthcare providers don’t trust 4what I tell them
  - o Healthcare providers don’t take into account my psychological problems
  - o Healthcare providers neglect some problems for others
  - o For some healthcare providers, I’m just a condition and not a person

**2.4. Situational factors**

- Out of routine
  - o Plan and organize travel
  - o Store medications when not at home
  - o Take medications when not at home
  - o Access to structures or equipment when not at home
  - o Pregnancy

- Other situational factors
  - o Changing physicians
  - o Organize my diet to accommodate other people
  - o Follow my diet in the presence of other people

**2.5. Financial factors**

## Box 3: Consequences of healthcare tasks imposed on patients in their daily lives

**3.1. Lack of adherence**

- Intentional non-adherence because of complexity
- Intentional non-adherence because of costs
- Non-intentional non-adherence and strategies not to forget treatment

**3.2. Impact on professional, social, family life, and leisure activities**

- Opportunity cost in professional life.
- Coping with absence from work.
- My healthcare activities interfere with my career (e.g., I didn’t get the job/promotion I wanted)
- Coping with judgment from others
- Treatment takes time/energy or requires precautions that interfere with family/friend commitments
- My healthcare activities interfere with my couple life
- Treatment takes time/energy or require precautions that interfere with leisure activities

**3.3. Emotional impact**

- Frustration because of not being able to do everything I want to
- Guilt associated with intentional non-adherence to treatment
- Treatment reminds me that I have a chronic condition

**3.4. Financial impact of healthcare tasks imposed on patients**

- Direct costs of treatment
- Indirect costs of treatment

## Additional files

Additional file 1:
**Questionnaire used on the website (English version).**
Additional file 2:
**Patient associations, physician organizations, and social media used for recruiting participants.**
Additional file 3:
**Extra tasks patients must perform because of their conditions (n = 1,053).**
Additional file 4:
**Factors related to the nature and frequency of imposed tasks that exacerbate the burden of treatment (n = 1,053).**
Additional file 5:
**Structural factors that exacerbate the burden of treatment (n = 1,053).**
Additional file 6:
**Personal factors that exacerbate the burden of treatment (n = 1,053).**
Additional file 7:
**Situational factors that exacerbate the burden of treatment (n = 1,053).**
Additional file 8:
**Consequences of healthcare tasks imposed on patients in their daily lives (n = 1,053).**
Additional file 9:
**Automatic textual analysis of English answers to open-ended questions (n = 308).**
Additional file 10:
**Automatic textual analysis of answers to open-ended questions in French and Spanish.**
Additional file 11:
**Odds ratios (with 95 % CI) for components of the burden of treatment elicited by patients in terms of presence of multimorbidity (adjusted for age, gender, educational level).**
Additional file 12:
**Odds ratios (with 95 % CI) for components of the burden of treatment elicited by patients in terms of age (adjusted for presence of multimorbidity, gender, educational level).**
Additional file 13:
**Odds ratios (with 95 % CI) for components of the burden of treatment elicited by patients in terms of educational level (adjusted for presence of multimorbidity, gender, age).**
Additional file 14:
**Odds ratios (with 95 % CI) for components of the burden of treatment elicited by patients in terms of gender (adjusted for presence of multimorbidity, educational level, age).**
