# Supplementary material for: Taxonomy of the burden of treatment: a multi-country web-based qualitative study of patients with chronic conditions
Source: BMC Med. 2015 May 14;13:115. doi: 10.1186/s12916-015-0356-x (PMC4446135; doi:10.1186/s12916-015-0356-x)
Supplement: Additional file 1: — Questionnaire used on the website (English version). [file 12916_2015_356_MOESM1_ESM.docx]

**Additional file 1: Questionnaire used on the website (English version)**

*How old are you?*  *|_ _ |*

*Gender? Male* □ *Female* □

*What is your* ***marital status****?*

- Married or civil union □
- Live in partner □
- Single □
- Divorced □
- Widow □

*Do you have an* ***informal caregiver*** *(such as a family member or a friend, who helps you with healthcare tasks without payment for the care given)?*

- Yes □
- No □

*(Conditional question) If you have, is it:*

- *A family member □*
- *A friend □*
- *Other ……………………*

*What is your* ***level of education****?*

- Primary and middle school □
- High School □
- College, University □

*In which* ***country*** *do you live?*  *……………………*

*Where do you go****most frequently****for medical consultations?*

- Public hospital □
- Private hospital □
- General practitioner’s clinic □
- Specialist’s clinic □

*Over the past 12 months, how many times were you hospitalized?* *|_ _ |*

*What are your main conditions (Multiple answers allowed)?*

Diabetes □

Thyroid disease or other endocrine disorders □

Lung diseases (for example: chronic bronchitis or asthma) □

High blood pressure □

Heart diseases (for example: myocardial infarction, heart failure, angina) □

Kidney diseases □

Gastrointestinal diseases (for example: ulcer, colitis, liver problems, gallbladder disease)□

Stroke or cerebrovascular disease □

Neurologic diseases (for example: Multiple sclerosis, Parkinson’s disease or epilepsy) □

Rheumatologic disease (for example: osteoporosis, arthritis or inflammatory polyarthropathies) □

Cancer or malignant blood diseases □

Depression, bipolar disorder, anxiety or other psychiatric diseases □

Vision problems (for example: glaucoma, cataract, macular degeneration) □

Hearing problems □

Skin diseases (for example: psoriasis) □

Infectious disease (for example: HIV illness, tuberculosis) □

Other disease: …………………………………………………………………………………………………

*For how long have you had at least one chronic condition? |_ _ |*

*Do you feel that you know enough about your conditions (for example: symptoms, disease progression...)?*

Not at all □ □ □ □ □ Very much

*Do you feel that you know enough about your treatments (for example: possible side effects, expected benefits, other treatment options...)?*

Not at all □ □ □ □ □ Very much

***The following question is crucial for our study. Take your time and feel free to write anything that crosses your mind. All comments will be taken into account.***

*Being a patient with chronic conditions can sometimes require a lot of time and effort, for example: 
 -   Visits to the doctor, arranging appointments...*

*-   Lab tests and other exams...*

*-   Taking medications everyday without forgetting them*

*-   Not being able to eat certain foods or drink alcohol, having to quit smoking...****All these things are very important but, sometimes, are also difficult to manage in everyday life. 

Think about what you do to take care of your health, and how this investment of time and effort can interfere with your everyday life.***

|  |
| --- |

*How many different****tablets, pills, puffs*** *do you take?* |_ _|

*per day / per week*

*How many shots do you have to take?* |_ _|

*per day / per week*

*How many****times****a day do you take medication?* |_ _|

*How long does it take you****to organize****your treatment? 
(for example: time required for sorting your pillboxes, prescriptions refills...)*

*|_ _| min per day*

*|_ _| min per week*

***Many patients find that some aspects of their treatment are too complex, limiting or time consuming.***

***Please think about these aspects of your treatment.***

*-* ***Taste, shape or size of tablets*** *and/or the* ***discomfort caused by injections*** *(for example, pain, bleeding, scars)*

*Could you tell us about how this impacts your everyday life?*

|  |
| --- |

*- The* ***number*** *medication intake per day*

*Could you tell us about how this impacts your everyday life?*

|  |
| --- |

*-* ***Remembering to take medication*** *and/or* ***managing*** *treatment when away from home (for example: sorting out pillboxes)*

*Could you tell us about how this impacts your everyday life?*

|  |
| --- |

- *Having to* ***follow specific procedures when taking medications*** *(for example, taking it at a specific time of the day or meal, not being able to do certain things after taking them like driving or lying down) or* ***storing them*** *(in their fridge…)*

*Could you tell us about how this impacts your everyday life?*

|  |
| --- |

- *Having to* ***visit doctors for medicine refills*** *and having to* ***go repeatedly to the pharmacy*** *to pick up the refills*

*Could you tell us about how this impacts your everyday life?*

|  |
| --- |

*How much time do you spend* ***"self-monitoring"*** *your condition or treatments (for example: self-measurement of blood pressure, keeping a diary)?*

*|_ _| min per day*

*|_ _| min per week*

*How many* ***different doctors*** *do you regularly see (for example, a GP, a cardiologist, a pulmonologist ...)?* | _ _ |

*In the past month, how many* ***medical appointments*** *did you attend (for example: doctor visits, blood tests or other exams...)?*  | _ _ |

***Many patients find that some aspects of their medical follow-up are too complex, limiting or time consuming.***

***Please think about these aspects of your follow-up.***

- *Frequency, time spent and/or inconvenience of* ***lab tests and other exams*** *(for example: scans, X-rays, blood tests…)*

*Could you tell us about how this impacts your everyday life?*

|  |
| --- |

- *Frequency, time spent and/or inconvenience of* ***self-monitoring*** *(for example, measuring blood sugar levels or blood pressure)*

*Could you tell us about how this impacts your everyday life?*

|  |
| --- |

- *Frequency, time spent and/or waiting* ***time of doctor visits,*** *including waiting time.*

*Could you tell us about how this impacts your everyday life?*

|  |
| --- |

- ***Fitting in appointments,*** *doctor visits and lab tests and* ***arranging transportation*** *(for example: scheduling and waiting for public transportation or ambulances, having to ask someone for a ride, parking problems…).*

*Could you tell us about how this impacts your everyday life?*

|  |
| --- |

***Many patients find that some aspects of their healthcare are too complex, limiting or time consuming.***

***Please think about these aspects of your healthcare.***

- ***Sorting out the paperwork*** *from health insurance companies, welfare organizations, hospitals and/or social care (for example: complete forms or administrative tasks to schedule appointments or to get reimbursements)?*

*Could you tell us about how this impacts your everyday life?*

|  |
| --- |

- ***The financial burden*** *associated with your healthcare (for example: out of pocket expenses or expenses not covered by insurance)?*

*Could you tell us about how this impacts your everyday life?*

|  |
| --- |

- ***Having to follow a specific diet****, not being able to eat certain foods, to drink alcohol or having to quit smoking?*

*Could you tell us about how this impacts your everyday life?*

|  |
| --- |

- *Following doctors advice to regularly* ***practice physical exercise****?*

*Could you tell us about how this impacts your everyday life?*

|  |
| --- |

- *How difficult it is to integrate healthcare needs* ***in your family, social or professional life*** *(for example: having to rely on others to do certain things, feeling uncomfortable taking medications in public, reconciling healthcare needs with culture and beliefs...)*

*Could you tell us about how this impacts your everyday life?*

|  |
| --- |

- ***“Looking after my health reminds me of my conditions”***

*Could you tell us about how this impacts your everyday life?*

|  |
| --- |

- *We wish to understand the difficulties patients face so they can take care of themselves. Some of these difficulties may be related to* ***the health system****(health insurance coverage, access to care close to home, hospital organization, health policy ...)*

*Could you tell us about how this impacts your everyday life?*

|  |
| --- |

*We have mentioned many things involved in your medical care you might find burdensome.****If other points were not mentioned or were insufficiently developed, take a few moments to share them with us:****Contrarily, if the questionnaire was sufficiently complete, please tell us so:*

|  |
| --- |

*Did someone help you complete the questionnaire?*

Yes □ No □

*(Conditional question) If someone did, who is it?*

- Your spouse/companion □
- A child □
- A friend □
- A healthcare professional □
- Other □

## *You might know others with treatment burden, tell them about this study!*

   Simply enter their **email address** to invite them to participate

|  |
| --- |
