# Supplementary material for: Taxonomy of the burden of treatment: a multi-country web-based qualitative study of patients with chronic conditions
Source: BMC Med. 2015 May 14;13:115. doi: 10.1186/s12916-015-0356-x (PMC4446135; doi:10.1186/s12916-015-0356-x)
Supplement: Additional file 2: — Patient associations, physician organizations, and social media used for recruiting participants. [file 12916_2015_356_MOESM2_ESM.docx]

**Additional file 2: Patient associations, physician organizations and social media used for recruitment of participants**

| **Patient associations that agreed invite their members to participate in the study** | ACS - Action contre les Spondylarthropathies  AFLAR - Association Française de Lutte Anti-Rhumatismale  AFS - Association France Spondylarthrite  Alliance du cœur  AMVF - Association des Malades des Vaisseaux du Foie  ANDAR - Association Nationale de Défense contre l'Arthrite Rhumatoïde  Associació Catalana de Fibrosi Quística  Association des sclérodermiques de France  Association Francophone pour Vaincre les Douleurs  Association Patients du Canada  Atrial fibrillation Association  Australian Patient Association  DEBRA International  EMSP - European Multiple Sclerosis Platform  Epilepsie France  FNETH - Federación nacional de enfermos y trasplantados hepáticos  Heart Sisters  HTAP France  Ligue Contre le Cancer  Lupus Europe  Lupus France  Pain Alliance Europe  Parenteral Nutrition-Down Under  PHA Europe  Renaloo  SOS hépatite  Wired4Life |
| --- | --- |
| **Physician organizations that distributed information via e-mail about the study** | Orphanet via their newsletter  Cochrane collaboration via the Consumer Network |
| **Social media** | Twitter  <https://twitter.com/BurdenTreatment>  Mayo clinic Social Media Health Network  <http://network.socialmedia.mayoclinic.org/discussion/helping-patients-bear-the-burden-of-treatment>  Facebook page  <https://www.facebook.com/pages/Burden-of-Treatment/366884696759186> |
