# Supplementary material for: Taxonomy of the burden of treatment: a multi-country web-based qualitative study of patients with chronic conditions
Source: BMC Med. 2015 May 14;13:115. doi: 10.1186/s12916-015-0356-x (PMC4446135; doi:10.1186/s12916-015-0356-x)
Supplement: Additional file 3: — Extra tasks patients must perform because of their conditions (n = 1,053). [file 12916_2015_356_MOESM3_ESM.docx]

**Additional file 3: Extra tasks patient must perform because of their conditions (n=1,053)**

| **Burden of treatment category** | **Example** | **Patients mentioning this burden**  **In total**  **- No. (%)** | **Patients mentioning**  **this burden spontaneously**  **- No. (%)*** |
| --- | --- | --- | --- |
| **Managing medications** | | | |
| **Prepare and take drugs correctly** | “That syringe is difficult to handle - if you want to take off the cap, then the syringe sometimes unscrews from the syringe and you have spoiled a 200 EUR treatment” | 237 (22) | 123 (12) |
| **Plan day accordingly to drug intakes** | “Everything has to be planned for, you can no longer be spontaneous and have to make sure you take your pills at the proper times or you end up suffering the consequences.” | 185 (18) | 108 (10) |
| **Following specific treatment precautions** | “One medication requires you do not eat for two hours before taking the drug or one hour after. I take this twice a day. Fitting this into a schedule especially when travelling or visiting or having company is very difficult.” | 291 (28) | 127 (12) |
| **Plan and organize refills** | “This means I have to be on constant watch that I do not let supplies get too” | 231 (22) | 51 (4.8) |
| **Store medications at home** | “Medication storage is a bit inconvenient since they're supposed to be away from heat and light, and not in the kitchen or bathroom where there's moisture. They do take up a lot of space because there are many different bottles.” | 111 (10) | 20 (1.9) |
| **Always have medication on person** | “I have to ensure I have a spare Kalydeco tablet with me at all times in case I am delayed and have to eat a meal away from home, as this has to be taken with a fatty meal.” | 111 (16) | 53 (5.0) |
| **Organizing and performing non-pharmacological treatment** | | | |
| **Access/use equipment** | “We have built a wheelchair friendly home so that I can hopefully still be independent in my future years.” | 35 (3.3) | 28 (2.6) |
| **Plan/perform physical therapy** | “They tell us to do physical therapy everyday; it’s tiring physically and mentally. Stretch up every day: when? In the evening I’m tired because of my day and my condition”** | 52 (4.9) | 38 (3.6) |
| **Condition or treatment follow-up** | | | |
| **Plan and organize self monitoring** | “I should test my blood every day. I do not. I go through phases where I test every day or every other day. And then sometimes I don't for weeks. It takes too long when I am running late for work in the morning.” | 10 (0.9) | 1 (0.1) |
| **Plan and organize lab tests** | “It’s a day off work (impact on me). At least I can book an appt for lab work but it’s still over an hour there. Hospital tests are good-about 3 hours start to finish. I usually don't plan anything else that day and make sure I have meals in the fridge the day before as I already know I won't have the energy to prep a meal” | 184 (17) | 66 (6.3) |
| **Precautions before/when performing tests** | “I have to lie down when I get blood taken. It's worse when I am fasting, which is usually the case. The lab nurses get frustrated because they only have one room at the lab with a bed. But if I don't tell them I need to lie down and I start to feel faint during the blood draw, they get mad that I didn't tell them.” | 36 (3.4) | 8 (0.8) |
| **Plan and organize doctor visits** | “That means that we have to manage our lives around the appointments. There is no coordination of the appointment arrangements. It appears that the relevant systems doe not talk with one another. This means that the patient remains responsible for managing arrangements and that places more stress on the overall situation.” | 428 (41) | 205 (19) |
| **Remember questions to ask the doctor** | “Trying to get a drs appointment can be difficult so I have to have prepared for the appointment with a list [of questions] of what is needed” | 8 (0.8) | 1 (0.1) |
| **Organize transportation** | “I have to book wheelchair taxis several hours in advance and can only book to get to the appointment because I don't know how long I will wait for the appointment after arriving. Then when I call a taxi at the end of the appointment I can have a long wait.” | 53 (5) | 11 (1.0) |
| **Lifestyle changes** | | | |
| **Force myself to eat some foods** | “Trying to eat a high calorie diet and maintain normal blood sugar levels is extremely difficult. (…) It is also difficult trying to eat when you don't always feel well.” | 23 (2.2) | 15 (1.4) |
| **Cut on some foods** | “I hate dieting, it depresses me, and I do not enjoy having a limited diet. It makes me feel punished. I don't smoke, but I do drink wine most evenings, so being told to quit drinking is very frustrating, even though I know that is good for me.” | 356 (34) | 215 (20) |
| **Plan and prepare meals** | “I always have to plan ahead & cook my own meals. This takes considerable time & energy.” | 80 (7.6) | 54 (5.1) |
| **Be careful of ingredients** | “Extremely time consuming - its constant. I have to read every package for ingredients. I can't simply enjoy eating out with friends.” | 65 (6.2) | 39 (3.7) |
| **Organize physical exercise** | “I don't do it [following medical advice to practice regular physical exercise]. I work full time and have two children under three. (…) The medical advice to exercise and eat only the correct things and don't work too hard and don't get stressed is pathetically unrealistic.” | 43 (4.1) | 38 (3.6) |
| **Perform some physical activities** | “[Doctors tell me] I should swim but I hate water !”** | 199 (19) | 70 (6.6) |
| **Give up some physical activities** | “I don't follow their advice [to give up some activities]. If I did I would not get out of the bed.” | 48 (4.6) | 16 (1.5) |
| **Change/organize my sleep schedule** | “Making sure I get at least 9 hours sleep a day, which may mean canceling plans or failing to work if I had a night of insomnia.” | 17 (1.6) | 14 (1.3) |
| **Give up smoking** | “I stopped smoking before transplantation. It’s been ten years and I still miss it”** | 37 (3.5) | 22 (2.1) |
| **Perform other lifestyle changes** | “I must avoid sunlight even though I need to walk daily for low impact exercise.” | 110 (10) | 80 (7.6) |
| **Organize formal caregiver care** | | | |
| **Organize formal caregiver care** | “But they [the nurses] kept coming to my home at eight in the morning, avoiding me to have any kind of love relationship and resulted into daily stress” ** | 8 (0.8) | 6 (0.6) |
| **Paperwork tasks** | | | |
| **Take care of administrative paperwork** | “This part of it drives me insane with frustration. I tend to ignore bills, paperwork and admin when I’m ill and end up in trouble with various faceless organizations screaming at me on the phone for late payments of incomplete paperwork. pah!” | 277 (26) | 25 (2.4) |
| **Organize medical paperwork** | “I keep all my medical tests/reports/radiology/referrals in a file - it's very time consuming recording this, but vital so I can follow any changes in test results. I can then see if anything is changing & needs to be actioned. I keep a spreadsheet & data chart.” | 51 (4.8) | 19 (1.8) |
| **Learning about the condition/treatment** | | | |
| **Learning about my condition or treatment** | “I have tried to learn about my health conditions and when available have taken courses on the condition. I read about the conditions and follow the doctors recommendations regarding tests and medication” | 79 (7.5) | 47 (4.4) |
| **Learn to navigate the healthcare system** | “It's very complex. you have to be experienced with the whole medical system and then know how to advocate for yourself and navigate through a very complicated process” | 64 (6.1) | 17 (1.6) |

*Spontaneously refers to patients mentioning the burden in the first broad open ended-question of the survey, prior to probes. **Translated from another language
