# Supplementary material for: Taxonomy of the burden of treatment: a multi-country web-based qualitative study of patients with chronic conditions
Source: BMC Med. 2015 May 14;13:115. doi: 10.1186/s12916-015-0356-x (PMC4446135; doi:10.1186/s12916-015-0356-x)
Supplement: Additional file 4: — Factors related to the nature and frequency of imposed tasks that exacerbate the burden of treatment (n = 1,053). [file 12916_2015_356_MOESM4_ESM.docx]

**Additional file 4: Factors related to the nature and frequency of imposed tasks that exacerbate the burden of treatment (n=1,053)**

| **Burden of treatment category** | **Example** | **Patients mentioning this burden**  **In total**  **- No. (%)** | | **Patients mentioning**  **this burden spontaneously**  **- No. (%)*** |
| --- | --- | --- | --- | --- |
| **Related to the nature of the tasks** | | | | |
| **Treatment characteristics (taste, shape, size of tablets, discomfort of injections** | “I sometimes struggle to swallow my omeprazole (the biggest pill I have to take) and I can only take certain brands acetaminophen (…) because some are too hard to swallow and my domperidone tastes really bad.” | | 331 (31) | 31 (2.9) |
| **Getting the right dose is difficult (addition of tablets or fraction of tablets)** | “I have to cut my tablets into quarters and add different dosages of the same medication to get the right amount”** | | 24 (2.3) | 9 (0.9) |
| **Appearance of medications can be confusing** | “Different makes of the same med can look different which with brain-fog can be confusing” | | 23 (2.2) | 4 (0.4) |
| **Treatment is for whole life** | “I will have to take medication for the rest of my life, there aren’t holidays for treatment”** | | 113 (11) | 88 (8.3) |
| **Medication side effects (real or imaginary)** | “(…) two screws inserted during patella tendon realignment. The screws went right through the bone in to the calf muscles.” | | 323 (31) | 174 (16) |
| **Discomfort associated with tests** | “Having blood drawn is often difficult. The vein in my arm often "hides", and can't be found at all, and blood has to be drawn from the back of my hand, which is very painful” | | 109 (10) | 23 (2.2) |
| **Discomfort associated with self-monitoring** | “I should test my blood every day. I do not (…). It hurts.” | | 46 (4.4) | 5 (0.5) |
| **Discomfort associated with consultations** | “The biggest problem I have is more the dignity of tests. When stripped for something like a heart echo, and other doctors walk into the room or you're not given a chance to cover yourself” | | 12 (1.1) | 3 (0.3) |
| **Time required to perform tasks** | | | | |
| **Time needed to take/organize treatment** | “Basic everyday treatments can sometimes be very time consuming. On a working day it would mean I have to get up extra early to complete everything before work” | | 97 (9.2) | 68 (6.4) |
| **Time needed for tests** | “I am required to get monthly as well as 3-monthly blood tests. This requires organization and time.” | | 160 (15) | 23 (2.2) |
| **Time needed for self monitoring** | “Wearing a heart rate monitor and logging activities and heart rate over a full day is time consuming and annoying. Writing that down consistently takes WAY more time than I would have imagined.” | | 50 (4.7) | 10 (0.9) |
| **Time needed for refills** | “Repeated visits to pharmacists to make sure I have the correct meds take a considerable amount of time, especially as (…) pharmacists take considerable time to either dispense the prescription or have long queues to actually collect the medication.” | | 96 (9.1) | 16 (1.5) |
| **Time needed for doctor visits** | “For many appointments, you must leave time for: getting to the appointment, finding parking, waiting for the appointment, seeing the doctor, getting back home. That can easily wipe out a morning or an afternoon” | | 206 (19) | 48 (4.5) |
| **Related to the number/frequency of the tasks** | | | | |
| **Number/frequency of drug intakes** | “Medications multiple times a day, and supplements multiple times a day, all scheduled so as not to interfere with each other and to meet different requirements” | | 298 (28) | 239 (23) |
| **Number/frequency of refills** | “I go to the pharmacy so often that they know me by name! Not a good thing.” | | 125 (12) | 39 (3.7) |
| **Number/frequency of tests** | “For annual review, it would be much better if all investigations could be ordered for the same day, then one visit and it is all done. But the hospital won't do that, so I have to make maybe 4 or 5 trips to different hospitals every year for this.” | | 270 (26) | 224 (21) |
| **Number/frequency of self monitoring acts** | “Some days, it becomes a matter of why am I doing this every day. No one will look at it. Why do they want it? Why am I spending so much time trying to be thorough when nothing will change?” | | 48 (4.5) | 33 (3.1) |
| **Number/frequency of doctor visits** | “[My condition/treatment] require a lot of medical follow ups, some every 4 months, some every year. Over the year, I average 1 to 2 appointments a month.” | | 274 (26) | 226 (21) |

*Spontaneously refers to patients mentioning the burden in the first broad open ended-question of the survey, prior to probes. **Translated from another language
