# Supplementary material for: Taxonomy of the burden of treatment: a multi-country web-based qualitative study of patients with chronic conditions
Source: BMC Med. 2015 May 14;13:115. doi: 10.1186/s12916-015-0356-x (PMC4446135; doi:10.1186/s12916-015-0356-x)
Supplement: Additional file 5: — Structural factors that exacerbate the burden of treatment (n = 1,053). [file 12916_2015_356_MOESM5_ESM.docx]

**Additional file 5: Structural factors that exacerbate the burden of treatment (n=1,053)**

| **Burden of treatment category** | **Example** | **Patients mentioning this burden**  **In total**  **- No. (%)** | | **Patients mentioning**  **this burden spontaneously**  **- No. (%)*** |
| --- | --- | --- | --- | --- |
| **Access to resources** | | | | |
| Pharmacy doesn’t have my medication in stock | “It takes such a time to replace them-if for some reason the pharmacist does not have them in stock this can be extremely stressful.” | 37 (3.5) | | 6 (0.6) |
| Access to lab test results | “Doing tests is not fun. But it gets painful when results are not commented. Information is filtering through in dribs and drabs from doctors.”** | 18 (1.7) | | 5 (0.5) |
| Access the right healthcare provider | “Finding a family doctor that can and will take on a patient with multiple co-morbidities is a major problem. Then finding one that is "smart" and able to cope with your conditions is even harder.” | 120 (11) | | 41 (3.9) |
| Distance from healthcare facilities | “I am able to drive but the distance and traffic factor into my comfort level for sure.” | 286 (27) | | 91 (8.6) |
| Difficulty planning last minute consultations | “Most doctors schedule a few weeks to a couple of months in advance, so it is hard to manage when you might NEED to see your doctor ahead of time.” | 28 (2.7) | | 10 (0.9) |
| **No coordination between care providers** | | | | |
| No coordination between care providers | “That would be great if I could get any 2 doctors or specialist to agree on an activity that doesn't risk permanent ongoing damage” | | 100 (9.5) | 39 (3.7) |
| **Healthcare center problems** | | | | |
| Wait times | “Doctors appointments (…) are sometimes stressful due to the wait in the surgery [doctors’ offices] when appointments are running late - as I run my own business I am also a busy person and regret the time wasted” | | 267 (25) | 36 (3.4) |
| Parking near healthcare facilities | “The car parking is so expensive at the hospital I am forced to park illegally.” | | 101 (9.6) | 10 (0.9) |
| **Research** | | | | |
| There is not enough research on my condition | “There is not enough research being done (…). There are no medications or devices approved for treating it. There are no accepted clinical guidelines for treatment. Everything is a stab in the dark. The biggest burden for me” | | 12 (1.1) | 3 (0.3) |
| **Media coverage** | | | | |
| Insufficient or inadequate media coverage of my condition | “My biggest problems at the moment are the media in my country, who are putting pressure on disabled people by characterizing us as "scroungers" who are really all fit to work. This stigma is difficult to bear.” | | 4 (0.4) | 0 (0.0) |

*Spontaneously refers to patients mentioning the burden in the first broad open ended-question of the survey, prior to probes. **Translated from another language
