# Supplementary material for: Taxonomy of the burden of treatment: a multi-country web-based qualitative study of patients with chronic conditions
Source: BMC Med. 2015 May 14;13:115. doi: 10.1186/s12916-015-0356-x (PMC4446135; doi:10.1186/s12916-015-0356-x)
Supplement: Additional file 6: — Personal factors that exacerbate the burden of treatment (n = 1,053). [file 12916_2015_356_MOESM6_ESM.docx]

**Additional file 6: Personal factors that exacerbate the burden of treatment (n=1,053)**

| **Burden of treatment category** | **Example** | **Patients mentioning this burden**  **In total**  **- No. (%)** | | **Patients mentioning**  **this burden spontaneously**  **- No. (%)*** |
| --- | --- | --- | --- | --- |
| **Beliefs** | | | | |
| **I’m anxious about performing tests and their results** | “It's a real stress that repeats every six months (…). A month before you think about it, during it it's terrible, and afterwards you never your results and even more complementary tests.” | | 126 (12) | 16 (1.5) |
| **I believe that some consultations are useless** | “I think it's ridiculous that I have to do follow-ups every month when my doctor doesn't appear to know much about my disease and definitely doesn't listen to me when I'm telling him how I'm doing and doesn't answer questions” | | 20 (1.9) | 4 (0.4) |
| **I believe that some follow-up tasks are useless** | “You are asked to keep track of all of this, and then when you bring it in to your appointment, the doctor barely glances at it.” | | 60 (5.7) | 11 (1) |
| **I believe that my treatment is inefficient** | “Treatment is a burden, but worse: it doesn’t work” | | 46 (4.4) | 34 (3.2) |
| **I feel dependant on my treatment** | “Difficulty is to accept that, although I’m young, I’m dependant on medications to live.” ** | | 39 (3.7) | 14 (1.3) |
| **My treatment conflicts with some of my religious beliefs** | “I can’t follow all of my religious tasks, especially fasting as my urea gets higher when I don’t drink” ** | | 2 (0.2) | 0 (0) |
| **Relationships with others (except: healthcare providers)** | | | | |
| **I feel that I’m a burden for others** | “Having to rely on family for help is very difficult and makes me feel like a burden” / “I feel inadequate and that I'm not doing my share” | | 158 (15) | 33 (3.1) |
| **My loved ones overdo things/ impose unnecessary precautions** | “Having family members tell me - stop doing this, or you'll get tired - that bothers me. I think I know when I'm tired. They don't realize that they make it worse by doing this (…) I want to be able to enjoy the "up" days, without constantly being told "slow down, you need to rest”” | | 28 (2.6) | 5 (0.5) |
| **My loved ones don’t help me with my condition/treatment** | “In my couple, I live my condition alone. My husband doesn’t care at all. Sometimes, I find that hard.” ** | | 15 (1.4) | 5 (0.5) |
| **I hide my condition or treatment from others** | “Cystic fibrosis makes you sweat more and so I feel I have to always wear tops to try and hide this.” | | 138 (13) | 21 (2) |
| **I have to regularly explain my conditions to others** | “Can appear to be anti social. Having to explain why you can't eat or drink certain things can be tiring.” | | 55 (5.2) | 11 (1) |
| **Seeing other patients reminds me of what could happen to me in the future** | “Even then, sometimes I will get stuck in line behind someone getting blood work for a transplant - I feel glad I don't need a transplant, but really, that shouldn't be handled at the walk in lab.” | | 6 (0.6) | 2 (0.2) |
| **Relationships with healthcare providers** | | | | |
| **My physicians don’t know about my condition/treatment** | “One neurologist even insisted that pills couldn't help me (…), and that exercise would cure me. The level of ignorance is quite astounding (…). I find it very frustrating to keep getting it, since they shouldn't be giving advice about an illness which they do not understand at all.” | | 82 (7.8) | 41 (3.9) |
| **My physician doesn’t take into account my context** | “Teach doctors that one way is NOT good for everyone. Individual care is vital as no two people are the same.” | | 46 (4.4) | 20 (1.9) |
| **Healthcare providers don’t explain things to me** | “All is about explaining. If doctors took time to explain why they prescribed this medication, maybe it would be less a burden” (translated) | | 71 (6.7) | 25 (2.4) |
| **I feel that healthcare providers don’t trust what I tell them** | “It took me three years to convince a single medical professional that something was actually wrong with my knee, before I was finally told that I had arthritis. During that time I struggled with depression and worse, because I was so rudely dismissed.” | | 44 (4.2) | 16 (1.5) |
| **Healthcare providers don’t take into account my psychological problems** | “It's like my physical health is more important that my mental health. They don't care that I'm in a bad mood as long as they can save my life. But what is my life worth if I'm unhappy. » | | 29 (2.7) | 7 (0.7) |
| **Healthcare providers neglect some problems for others** | “I see a number of specialists who have knowledge of one or two organ systems and are very good at managing diseases affecting those systems, but fail miserably to effectively manage other problems. As a result, I almost don’t take care of my other health problems.”** | | 6 (0.6) | 3 (0.3) |
| **“For some healthcare providers, I’m just a condition and not a person”** | “We as patients with chronic conditions are sometimes treated poorly by doctors and other healthcare members as drug seekers (…). I was horrified by the way I've been treated.” | | 54 (5.1) | 11 (1) |

*Spontaneously refers to patients mentioning the burden in the first broad open ended-question of the survey, prior to probes. **Translated from another language
