# Supplementary material for: Taxonomy of the burden of treatment: a multi-country web-based qualitative study of patients with chronic conditions
Source: BMC Med. 2015 May 14;13:115. doi: 10.1186/s12916-015-0356-x (PMC4446135; doi:10.1186/s12916-015-0356-x)
Supplement: Additional file 7: — Situational factors that exacerbate the burden of treatment (n = 1,053). [file 12916_2015_356_MOESM7_ESM.docx]

**Additional file 7: Situational factors that exacerbate the burden of treatment (n=1053)**

| **Burden of treatment category** | **Example** | **Patients mentioning this burden**  **In total**  **- No (%)** | | **Patients mentioning**  **this burden spontaneously**  **- No (%)*** |
| --- | --- | --- | --- | --- |
| **Out of routine** | | | | |
| **Plan and organize travels** | “Remember to take enough for total time I am going to be away when traveling. Get pharmacist to dispense extra. Take original pharmacy bottles when crossing borders” | | 198 (19) | 66 (6.3) |
| **Store medications when not at home** | “If I fly abroad, all my hand baggage is taken up by my prescription supplies. This can be a problem if going for a two week holiday due to the restriction on fluid volumes that can be carried into the cabin. It also means I can't take any other essential hand baggage items” | | 134 (13) | 21 (2) |
| **Take medications when not at home** | “The hardest thing is dealing with medications when changing time zones”  “Remembering to take the pills when out (…) because I don't have the same memory triggers when I'm outside of my normal daily routine” | | 132 (12) | 23 (2.2) |
| **Access to structures or equipment when not at home** | “Also getting access to a fridge is a difficulty (…) it effectively means we can't go camping any more. Some hotels are not good at allowing access to a fridge for medication. It is not nice to have to ask for medication in reception” | | 51 (4.8) | 23 (2.2) |
| **Pregnancy** | “Let me tell you about pregnancy and type 2 diabetes! It is comical. Doctors appointments every two weeks between the gynecologist and the endocrinologist, 6 blood sugar readings and 4 insulin injections per day, the hematomas you can get when you have the bad luck of getting your vein pierced through”** | | 7 (0.7) | 4 (0.4) |
| **Other situational factors** | | | | |
| **Changing physicians** | “I also used to see an orthopedist, because (…) they can prescribe physiotherapy and I respond well to that as a treatment option, but unfortunately my doctor left the practice and her replacement isn't comfortable with chronic pain management.” | | 9 (0.8) | 5 (0.5) |
| **Organize my diet in function of other people** | “You have to tell you hosts about your specific diet which is also a burden to them. When you eat with other people, you always have to choose a restaurant when you can also eat. It is not nice to impose such things to other people.”** | | 15 (1.4) | 6 (0.6) |
| **Follow my diet in presence of other people** | “I cannot share much food with others, have to bring my own food, must deal with the hassle of having to explain to others why I cannot eat whatever I want, have the stigma of feeling weird and different from other people” | | 128 (12) | 45 (4.3) |

*Spontaneously refers to patients mentioning the burden in the first broad open ended-question of the survey, prior to probes. **Translated from another language
