# Supplementary material for: Taxonomy of the burden of treatment: a multi-country web-based qualitative study of patients with chronic conditions
Source: BMC Med. 2015 May 14;13:115. doi: 10.1186/s12916-015-0356-x (PMC4446135; doi:10.1186/s12916-015-0356-x)
Supplement: Additional file 8: — Consequences of healthcare tasks imposed on patients in their daily lives (n = 1,053). [file 12916_2015_356_MOESM8_ESM.docx]

**Additional file 8: Consequences of healthcare tasks imposed to patients on their daily lives (n=1053)**

| **Burden of treatment category** | **Example** | **Patients mentioning this burden**  **In total**  **- No (%)** | | **Patients mentioning**  **this burden spontaneously**  **- No (%)*** |
| --- | --- | --- | --- | --- |
| **Lack of adherence** | | | | |
| **Intentional non-adherence because of complexity** | “I decided early on that I just couldn't handle the shots in my belly because my pants waistband would just irritate the injection site reaction and cause an endless, low-level misery...so I just stopped taking that shot, and settled on a 6-day-a-week schedule, giving myself a day "off". | | 38 (3.6) | 12 (1.1) |
| **Intentional non-adherence because of costs** | “I cannot afford to go to doctor visits every month, or every two months to refill my pain medications. I have had to stop taking my thyroid medications because I have no insurance or money to pay.” | | 26 (2.5) | 6 (0.6) |
| **Non-intentional non-adherence and strategies not to forget** | “Also dealing with remembering to change the fentanyl patch and what side it was last on etc is taxing and requires a calendar nearby for noting these changes” | | 393 (37) | 172 (16) |
| **Impact on professional, social, family life and leisure activities** | | | | |
| **Opportunity cost in professional life. Coping with absence from work** | “My hospital is very exhaustive with testing (…) All of these are within work hours so I always have to take time off work. In addition, all of the communication with services has to take place while I'm at work.” | | 266 (25) | 144 (14) |
| **My healthcare activities interfere with my career (e.g. I didn’t get the job/promotion I wanted)** | “I gave up my career in chemistry due to not being able to cope with the flare ups. It has had a devastating effect on my career and difficulty to find work in areas that I wish to.” | | 140 (13) | 73 (6.9) |
| **Coping with judgment from others** | “[When I] use my blue parking badge, I often get accused of abusing the system just because I don't have a wheelchair” | | 217 (21) | 47 (4.4) |
| **Treatment takes time/energy or require precautions that interfere with family/friends commitments** | “One doctor suggested my problems would be solved if I cycled an extra 5km every day and gave up my family time on Sundays to exercise instead (…) These suggestions seemed arbitrary and unhelpful, and I must admit I disregarded them.” | | 171 (16) | 91 (8.6) |
| **My healthcare activities interfere with my couple life** | “It is always a bit embarrassing to go to the bathroom with your purse when you're out with your friends or on a date, it looks like "she’s having her period or that girl only thinks about making herself up" whereas actually it's for an injection.”** | | 22 (2.1) | 11 (1) |
| **Treatment takes time/energy or require precautions that interfere with leisure activities** | “I no longer (…) spend time participating in leisure or recreation. Some of the medical stuff fits into the time those things would take, some gets done instead of taking a lunch break at work, the rest just doesn't get done.” | | 117 (11) | 48 (4.5) |
| **Emotional impact** | | | | |
| **Guilt associated with intentional non adherence to treatment** | “Every day I stick needles into my stomach and think about all the things I could have done to avoid the disease and all the things I should do but don't to lessen its impact on my life.” | | 48 (4.5) | 8 (0.8) |
| **Treatment reminds me that I have a chronic condition** | “Thinking of all things I can’t do, for example driving a motorcycle, remind me that I’m sick” | | 225 (21) | 48 (4.5) |
| **Financial impact of healthcare tasks imposed to patients** | | | | |
| **Direct costs of treatment** | “The costs of special equipment and special food, the cost of exercise programs, PT, OT, special shoes, a wheelchair accessible home with high property taxes, medication costs that are not covered, and the transportation to various appointments all take a big chunk out of income.” | | 347 (33) | 67 (6.3) |
| **Indirect costs of treatment** | “I lose income if I do not work. I have no sick days or vacation days. I am a sole proprietor of small business open 7 days a week so if I have to take time off work that means I lose money for that day.” | | 120 (11) | 23 (2.2) |

*Spontaneously refers to patients mentioning the burden in the first broad open ended-question of the survey, prior to probes. **Translated from another language
