# Supplementary material for: Taxonomy of the burden of treatment: a multi-country web-based qualitative study of patients with chronic conditions
Source: BMC Med. 2015 May 14;13:115. doi: 10.1186/s12916-015-0356-x (PMC4446135; doi:10.1186/s12916-015-0356-x)
Supplement: Additional file 9: — Automatic textual analysis of English answers to open-ended questions (n = 308). [file 12916_2015_356_MOESM9_ESM.docx]

**Additional file 9: Results of automatic textual analysis for English answers to open-ended questions (n=308).** Words presented are words strongly associated with each class (chi-square>50) in descending order. Bold words are words most commonly used by participants. * Drug names were changed to international non-proprietary names

| **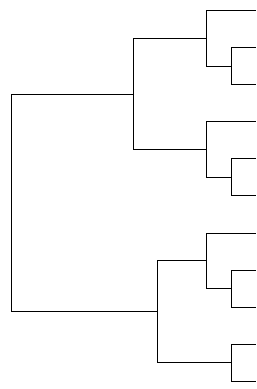** | **Tests and exams** | **Blood-test**; **wait**; lab; visit; pressure; appointment; scan; blood work; **time** |
| --- | --- | --- |
|  | **Organize appointments; transportation** | **Appointment**; come; **wait**; arrange; arrive; **schedule**; park |
|  | **Refills** | **Pharmacy**; refill; **prescription**; pick; call; order; mail; fax; email; request |
|  | **Store ; organize during travels** | **Supply**; bag; carry; fridge; bottle; mix; connect; **travel**; container; airport; pump; hotel; original; hook; **plan**; store; kitchen; infuse; luggage; nebulizer; oversea |
|  | **Side effects, characteristics of drugs** | **Side-effect**; injection; bruise; nausea; site; sting; painful; swallow; bee; inject; enoxaparin*; amitriptyline; cause; swell |
|  | **Management of medications** | **Pill**; stomach; breakfast; tablet; **morning**; **remember**; **medication**; taste; forget; empty; night; eat; alarm; dose; day |
|  | **Lifestyle changes (diet and exercise)** | **Exercise**; diet; **food**; alcohol; **eat**; weight; walk; smoke; rehab; calorie; appetite; healthy; gym; joint; arthritis; drink; cook |
|  | **Relationships with others (family friend)** | **Family**; member; old; **feel**; **friend**; **life** |
|  | **Relationships with others (society)** | **Condition**; chronic; understand; **health**; **person**; mental; treat; patient; management; fear; society |
|  | **Paperwork** | Disability; assistance; **cover**; **insurance**; benefit; medicare; apply; approve; patience; appeal; company; qualify; paperwork |
|  | **Financial costs** | **Insurance**; bill; **pay**; expense; cover; pocket; income; claim; government; **cost**; dollar; financial; coverage; thousand; park |
