# Supplementary material for: Taxonomy of the burden of treatment: a multi-country web-based qualitative study of patients with chronic conditions
Source: BMC Med. 2015 May 14;13:115. doi: 10.1186/s12916-015-0356-x (PMC4446135; doi:10.1186/s12916-015-0356-x)
Supplement: Additional file 10: — Automatic textual analysis of answers to open-ended questions in French and Spanish. [file 12916_2015_356_MOESM10_ESM.docx]

**Additional file 10a: Results of automatic textual analysis for answers to open ended questions in French (n=690).**

| 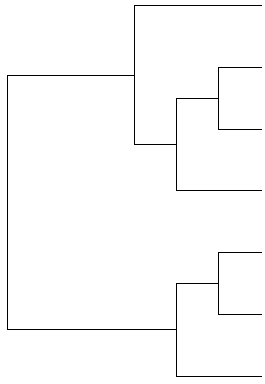 | **Visits, tests and refills** |
| --- | --- |
|  | **Secondary effects** |
|  | **Medication management (mainly strategies not to forget)** |
|  | **Self monitoring** |
|  | **Accept condition and relationships with family** |
|  | **Lifestyle changes** |
|  | **Paperwork and reimbursments** |

**Additional file 10b: Results of automatic textual analysis for answers to open ended questions in Spanish (n=42).**

| 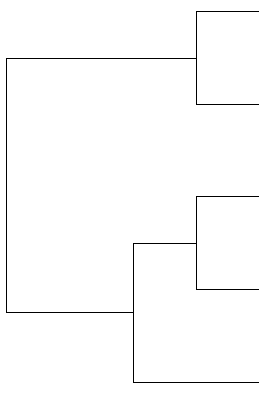 | **Paperwork and reimbursements** |
| --- | --- |
|  | **Family support, professional impact** |
|  | **Doctor visits, planning organization** |
|  | **Tests and self monitoring** |
|  | **Medication management** |
