# Supplementary material for: Taxonomy of the burden of treatment: a multi-country web-based qualitative study of patients with chronic conditions
Source: BMC Med. 2015 May 14;13:115. doi: 10.1186/s12916-015-0356-x (PMC4446135; doi:10.1186/s12916-015-0356-x)

**Additional file 11a: Odds ratios (with 95% CI) for components of the burden of treatment elicited by patients in terms of presence of multimorbidity (defined as patients with 2 or more chronic conditions) (adjusted for age, gender, educational level).** Higher OR indicates that patients with >2 chronic conditions elicited the burden more often than those with one chronic condition.


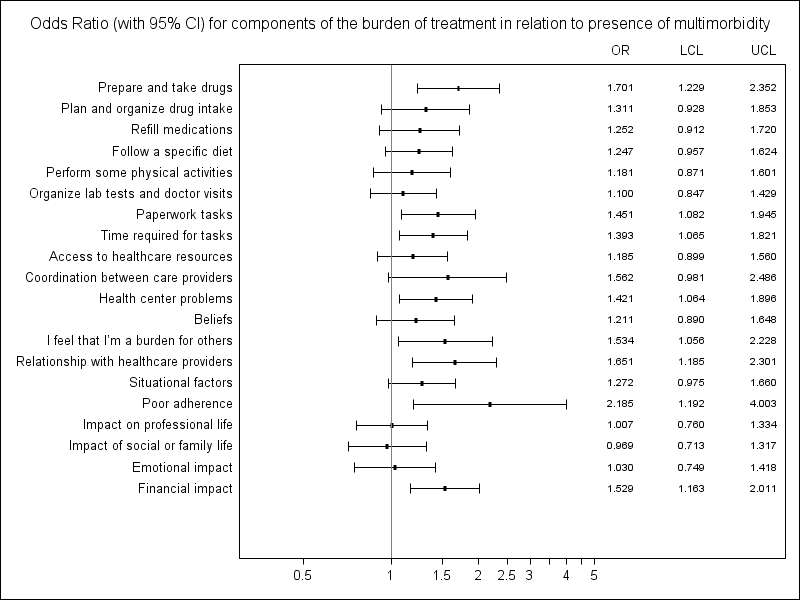


**Additional file 11b: Odds ratios (with 95% CI) for components of the burden of treatment elicited by patients in terms of presence of multimorbidity (defined as patients with 3 or more chronic conditions) (adjusted for age, gender, educational level).** Higher OR indicates that patients with >3 chronic conditions elicited the burden more often than those with one chronic condition.


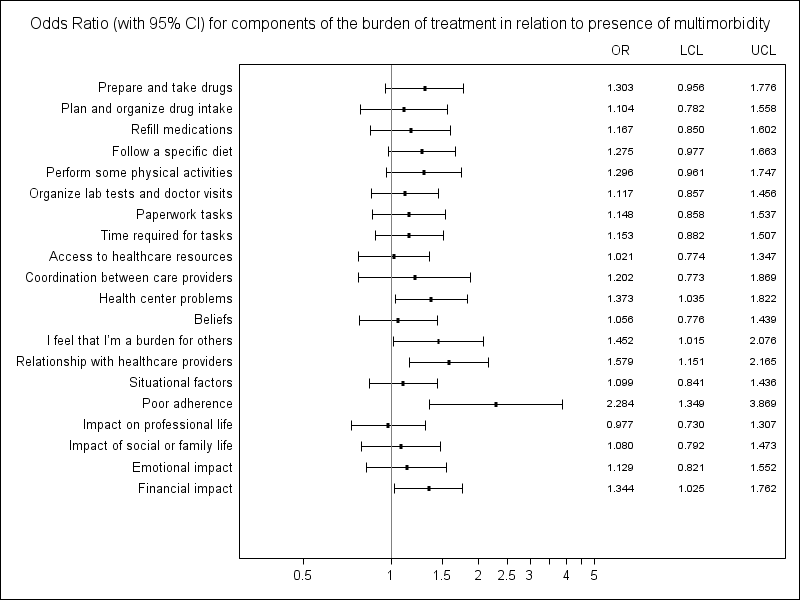

Supplement: Additional file 11: — Odds ratios (with 95 % CI) for components of the burden of treatment elicited by patients in terms of presence of multimorbidity (adjusted for age, gender, educational level). [file 12916_2015_356_MOESM11_ESM.docx]
