# Supplementary material for: Taxonomy of the burden of treatment: a multi-country web-based qualitative study of patients with chronic conditions
Source: BMC Med. 2015 May 14;13:115. doi: 10.1186/s12916-015-0356-x (PMC4446135; doi:10.1186/s12916-015-0356-x)

**Additional file 12: Odds ratios (with 95% CI) for components of the burden of treatment elicited by patients in terms of age (adjusted for presence of multimorbidity, gender, educational level).** Higher OR indicates that patients >50 years old elicited the burden more often than those < 50 years old.


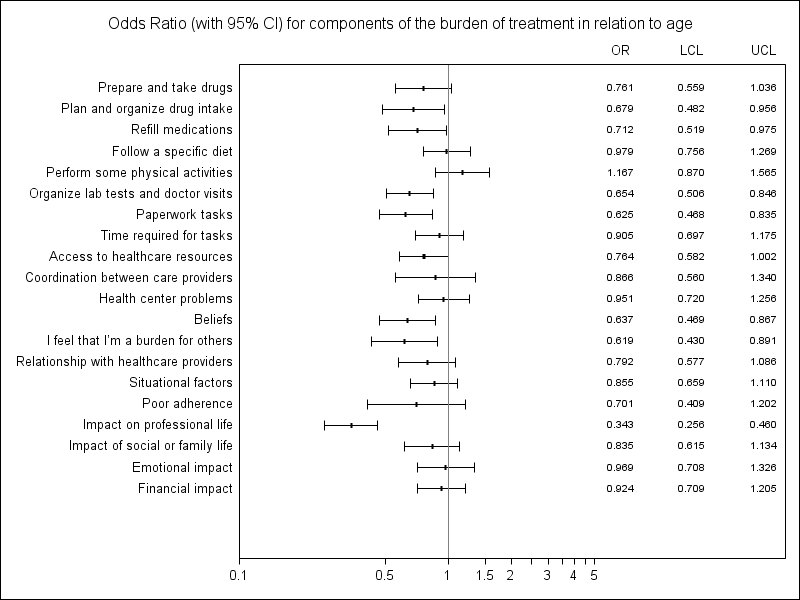

Supplement: Additional file 12: — Odds ratios (with 95 % CI) for components of the burden of treatment elicited by patients in terms of age (adjusted for presence of multimorbidity, gender, educational level). [file 12916_2015_356_MOESM12_ESM.docx]
