# Supplementary material for: Taxonomy of the burden of treatment: a multi-country web-based qualitative study of patients with chronic conditions
Source: BMC Med. 2015 May 14;13:115. doi: 10.1186/s12916-015-0356-x (PMC4446135; doi:10.1186/s12916-015-0356-x)

**Additional file 13: Odds ratios (with 95% CI) for components of the burden of treatment elicited by patients in terms of educational level (adjusted for presence of multimorbidity, gender, age).** Higher OR indicates that patients with higher educational level elicited the burden more often than those with lower educational level.


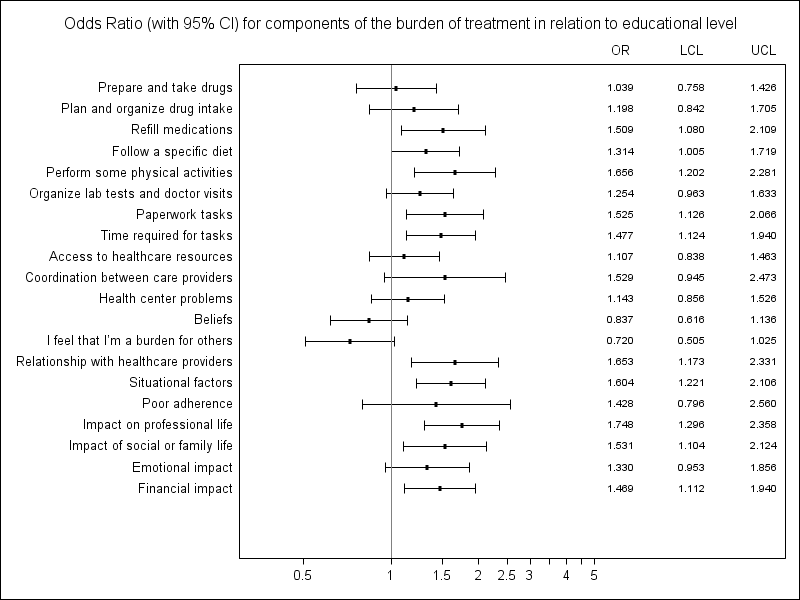

Supplement: Additional file 13: — Odds ratios (with 95 % CI) for components of the burden of treatment elicited by patients in terms of educational level (adjusted for presence of multimorbidity, gender, age). [file 12916_2015_356_MOESM13_ESM.docx]
