# Supplementary material for: Taxonomy of the burden of treatment: a multi-country web-based qualitative study of patients with chronic conditions
Source: BMC Med. 2015 May 14;13:115. doi: 10.1186/s12916-015-0356-x (PMC4446135; doi:10.1186/s12916-015-0356-x)

**Additional file 14: Odds ratios (with 95% CI) for components of the burden of treatment elicited by patients in terms of gender (adjusted for presence of multimorbidity, educational level, age).** Higher OR indicates that female participants elicited the burden more often than male participants.


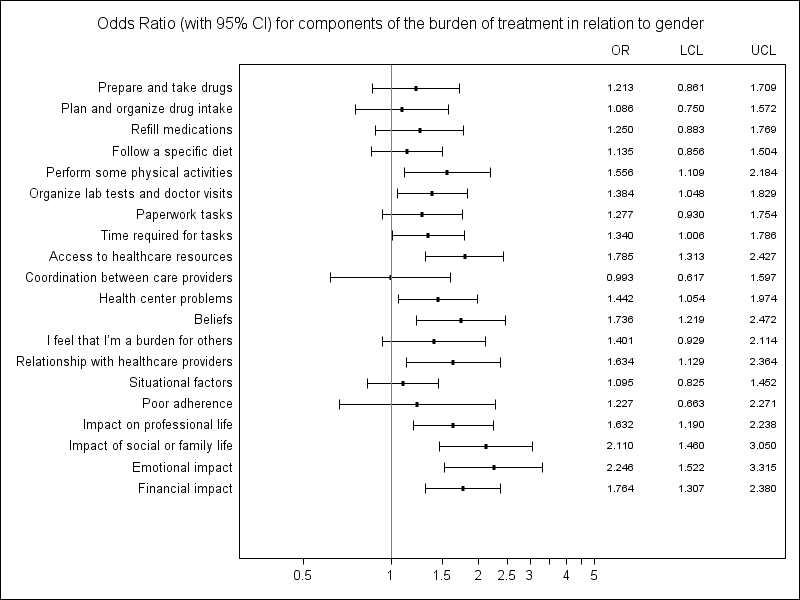

Supplement: Additional file 14: — Odds ratios (with 95 % CI) for components of the burden of treatment elicited by patients in terms of gender (adjusted for presence of multimorbidity, educational level, age). [file 12916_2015_356_MOESM14_ESM.docx]
